# Mixed-method study on the association between inclusion to conditional cash transfer program and the multiple faces of malnutrition in children and adolescents aged 3 to 19 years: a school-based evidence from Caraga Region, the Philippines

**DOI:** 10.1186/s12887-023-04438-8

**Published:** 2023-12-13

**Authors:** Deborah Jael Herrera, Miraluna L. Herrera, Donnacham Amora, Sherlyn Bas, Caryl Aya Miranda, Guido Van Hal

**Affiliations:** 1 Family Medicine and Population Health Department, Faculty of Medicine and Health Sciences, University of Antwerp, Antwerp, Belgium; 2 Action for Greater Advancement of Knowledge (AGAK) Center, Caraga State University, Agusan del Norte, Butuan City, Philippines; 3 Department of Medical Technology, Agusan del Norte, Butuan Doctors College, Butuan City, Philippines

**Keywords:** Mixed-method, Conditional cash transfer, Malnutrition, Overnutrition, Unintended consequences, Adaptive approaches

## Abstract

**Background:**

This study aimed to investigate the association between inclusion to *Pantawid Pamilyang Pilipino Program* (4Ps), a CCT program in the Philippines, and malnutrition in children and adolescents and examine the perceptions and experiences of parents and other stakeholders on how 4Ps influenced child nutrition.

**Methods:**

A concurrent mixed-method study was conducted in the Caraga Region, Philippines. Quantitative data from 5541 children and adolescents aged 3 to 19 were analyzed using multilevel mixed-effect logistic models. To allow in-depth understanding of the programmatic components that support the findings from the quantitative study, eight focused group discussions (FGDs) were concurrently conducted, cumulating 33 participants, including 4Ps parents, school coordinators/teachers, and school nurses. A constructivist grounded theory approach was used for analysis, and joint displays were employed to integrate quantitative and qualitative results.

**Results:**

Quantitative findings revealed high rates of malnutrition, with significant rates of stunting (12.0%), wasting/thinness (9.4%), and overweight/obesity (16.4%) among children and adolescents. 4Ps beneficiaries had higher odds of stunting and overweight/obesity compared to non-4Ps beneficiaries (AOR = 1.43, 95%CI: 1.08–1.91; AOR = 1.21, 95%CI: 1.01–1.45, respectively). However, no significant association was observed between inclusion to 4Ps and concurrent stunting and wasting/thinness or overweight/obesity (AOR = 1.05, 95%CI: 0.72–1.55). Geographic variations were observed, with 4Ps children in Agusan del Sur having lower odds of stunting than those in Agusan del Norte (AOR = 0.41, 95%CI: 0.23–0.71). Age and gender also showed significant associations with malnutrition. The qualitative analysis provided insights into the challenges contributing to malnutrition, including child labor, sickness, long distances to school, limited access to healthy food, and larger families. Unintended consequences such as cash card mismanagement, inappropriate cash grant allocation, and falsification of school attendance were identified. However, teachers and parents demonstrated resilience by implementing adaptive approaches to enhance child nutrition.

**Conclusions:**

While 4Ps beneficiaries exhibit higher odds of stunting and overweight/obesity, the program’s association with malnutrition was significantly influenced by geographic variations. It is crucial for social protection programs to prioritize comprehensive support strategies that effectively counter unintended consequences and challenges faced by beneficiaries and other stakeholders and address malnutrition in children and adolescents.

**Supplementary Information:**

The online version contains supplementary material available at 10.1186/s12887-023-04438-8.

## Background

Ending malnutrition in all its forms has long been at the core of the Sustainable Development Goals No.2 and the United Nations International Children’s Emergency Fund (UNICEF) since 1990 [1, 2]. The World Health Organization defines malnutrition as the overall nutritional status of an individual, including the undernutrition and overnutrition caused by deficiencies, excesses, or imbalance intake of energy and/or nutrients of an individual [3]. Several indicators of malnutrition in children and adolescents include existence of stunting, overweight, and its more severe form, obesity, and the co-existence of undernutrition and overnutrition [4, 5].

Despite the efforts to improve child nutrition, the State of the World’s Children report shows that malnutrition in children and adolescents remains to be a global burden, [6]. There are still 149 million children under 5 with stunting, and about 50 million with wasting in the 21st century [7]. Moreover, the prevalence of overweight and obesity, continues to rise in children and adolescents aged between 5 and 19 [2, 7]. Another evidence indicates that the double burden of malnutrition (DBM), which is the co-existence of under- and overnutrition, is highly prevalent in more than one-third of the 126 low-income and middle-income countries (LMICs) [4, 8]. In East Asia and Pacific Region, the Philippines is one of the top five countries with the highest prevalence of stunting, with nearly 1 in 3 children under 5 were stunted [9, 10]. Obesity and overweight, likewise, have been rapidly prevalent in Filipino children, particularly in children and adolescents aged between 5 and 19, and were forecasted to reach 30% by 2030 if no actions are taken [11]. The country’s malnutrition rates among children were found to be highly attributable to low-income households and thus, have resulted to urgent calls on the need to scale-up poverty reduction programs in addressing malnutrition, in all its forms [9].

Conditional cash transfer (CCT) programs, which are social protection programs, could provide opportunities to address malnutrition in children and adolescents living in low-income households [12]. CCT programs provide monetary transfers to the low-income and vulnerable households on the condition that they comply with a set of requirements [13, 14]. This provision requires beneficiary households to comply to education and health conditionalities, including health check-ups and growth monitoring of children as well as raising parental or legal guardian’s awareness and knowledge on child nutrition through family developmental sessions [9, 12, 15]. For over a decade, CCT programs have been promoted over in-kind transfer programs due to its cost-effectiveness on improving short-term and long-term behavioral and health outcomes in children and their families due to the conditionalities imposed to them [14, 16, 17].

Since 2008, the CCT program in the Philippines, known as the Pantawid Pamilyang Pilipino Program (4Ps), has been implemented nationwide and have been contributing significantly to the decrease in malnutrition rates in children under 5 [18]. The 4Ps has reached up to 300,000 households in its initial year and about 4.3 million active household beneficiaries in 2022, covering 26 per cent of the Philippines population [19]. During the pilot and initial implementation of 4Ps in the Philippines, strong evidence on the positive effects of 4Ps on nutrition among children living in low-income households were reported. These positive effects of 4Ps on nutritional outcomes were influenced by improved quantity and quality of food consumption in the household, access to health services and sanitations, and parental knowledge and attitudes on nutrition [9]. However, in 2018, the inadequate assistance due to the increased costs of living during inflations and the decreasing number of children covered by the program have resulted to a significant decrease in 4Ps’ impact on child nutrition [9, 18].

On another note, the prevalence and claims of irreversible consequences of malnutrition for children in their first 1,000 days of life (from conception to age two) were reported by numerous studies, which resulted to major policy shift focusing on early years [2, 20–23]. However, most recent studies have already disproven these irreversibility claims and have recognized the potential impacts of tailored interventions (e.g., growth- and nutrition-promoting interventions in school-aged children) to improve nutrition of children in their next 7,000 days of life (from aged 3 to 19) [24, 25]. The effects of early deprivation can still be intervened in the child’s later phase (catch-up growth) as it remains to be a crucial and sensitive phase that shape their development into adulthood [2]. Unfortunately, there is still a paucity of data available that explored the link between inclusion to 4Ps and malnutrition in children and adolescents aged between 3 and 19. Despite the strong evidence of 4Ps impact on child nutrition, most studies were only focused on one form of malnutrition (stunting), and were only limited to children under 5, particularly during their first 1,000 days of life [9]. To address these gaps, this study aimed to investigate the association between inclusion to 4Ps and various forms of malnutrition in children aged between 3 and 19, and to examine parents’ and teachers’ experiences and perspectives on how 4Ps has influenced child nutrition.

## Methods

### Study design

This study employed a concurrent mixed-method study design, which allowed the use of both quantitative and qualitative data and the integration of both findings to provide in-depth insights regarding the influence of *4Ps* on children’s nutritional outcomes.

### Study site

The study was conducted in Caraga Region, the Philippines, which includes the provinces of Agusan del Norte, Agusan del Sur, Surigao del Norte, and Surigao del Sur. In Agusan del Norte, the participating schools were predominantly situated in suburban and urban areas, providing convenient access to essential amenities like healthcare facilities, pharmacies, and main marketplaces. Conversely, in Agusan del Sur, Surigao del Sur, and Surigao del Norte, the representative schools were predominantly located in rural or coastal areas. Specifically, in Surigao del Norte, the participating schools were situated on islands, where access to healthcare facilities, pharmacies, and main marketplaces required at least a 30-minute boat journey. On the other hand, participating schools from Agusan del Sur were predominantly situated in rural areas, specifically mountainous settings.

### Quantitative study

#### Sampling and data collection

The study utilized a secondary school-based data on child nutrition, inclusion to *4Ps* and socio-demographic and -cultural factors collected between 2016 and 2021. These data were annually assessed by teachers and school nurses in compliance to the Department of Education Guidelines on the Preparation and Checking of School Forms [26]. Ten representative schools, comprising of five primary schools and five secondary schools, were selected from different provinces within the Caraga Region. These schools actively participated by providing the School Registry (referred to as School Form 1) and the learners’ health and nutritional assessment (referred to as School Form 8).

Inclusion to 4Ps (exposure of interest) of the children and adolescents were obtained from the City or Municipal Social Welfare and Development (CSWD/MSWD) Office by teacher-in-charge or 4Ps coordinator. Identification of children included in *4Ps* were validated through an official memorandum and list sent by the CSWD/MSWD to the 4Ps coordinators in schools. In this study, inclusion to 4Ps were categorized as non-4Ps or 4Ps child beneficiaries. Socio-demographic factors included were child’s sex (male/female), age (continuous) and locality (by province) while socio-cultural factors included were child’s religious affiliation, ethnicity, and mother tongue, which were all categorical nominal variables.

#### Outcome measures

Children’s nutritional status was assessed by school nurses using calibrated scales provided by the Department of Education and were recorded in ‘Learner’s Basic Health and Nutrition Report’ (school form 8) [27]. To account for variations influenced by age and sex, these measurements were converted to Z-scores using the AnthroPlus Software from the World Health Organization [28, 29]. The primary outcomes were stunting (yes/no), overweight or obesity (yes/no), and coexistence of stunting with wasting/thinness or overweight/obesity (yes/no). Stunting was defined as having a height-for-age Z-score (HAZ) below − 2SD, while overweight/obesity was determined as having a Body Mass Index -for-age Z-score (BAZ) of > 2SD for children 5 and below, and > 1SD for children above 5 years. Moreover, wasting/thinness was defined as having BMI-for-age (only for children above 5 years) and weight-for-height Z-scores (only for children below 5 years) of below − 2SD, respectively [30, 31]. Furthermore, the presence of DBM was assessed by considering the coexistence of stunting with either wasting/thinness or overweight/obesity.

### Data analysis

Quantitative analyses were carried out using the statistical package R (R Foundation for Statistical Computing©, version 4.0.3) [32]. Descriptive statistics were used to summarize the baseline characteristics of the study participants using frequency and proportion for categorical variables and mean and standard deviation (SD) for continuous variables. For the primary analyses, since schools were nested within province and children measured within the same year, mixed-effects logistic regression analysis was used to estimate the average impact of the 4Ps on child nutritional status while accounting for the random effects among representative schools and year measured [33]. This allowed us to account for unobservable unit-specific and time-specific confounders and for clustering effects (*i*.*e.*, pupils/students within the same schools and children measured within the same year) [34]. Akaike Information Criterion (AIC) was used as a model selection criterion with a *p*-value of < 0.05 used to define statistical significance. Multiple imputation was employed (m = 11, maxit = 20, and seed = 100) for data missing at random (MAR).

### Qualitative study

#### Recruitment of participants

Recruitment of study participants for the focused group discussion (FGD) was conducted using two different sampling methods: (1) Purposive sampling to recruit diverse stakeholders, including teachers, teacher-coordinators of 4Ps, and school nurses in different schools across Caraga Region, and (2) respondent-driven sampling (RDS), in which stakeholders were asked to recruit parents with 4Ps children and adolescents. Teachers or nurses who were newly employed within a period of three months were excluded from the study due to their limited familiarity or experience with the specific programmatic characteristics of 4Ps within the school or area. Moreover, in situations where the current school or district nurse or coordinator was newly appointed, the previous in-charge of 4Ps implementation in the school was considered.

### Participants and group composition for the qualitative study

The team identified teachers that could assist during the recruitment of parents of 4Ps children and provided an orientation about the purpose of the study and the target study participants for the FGDs. Sampling was done by school and school division, with 1 to 2 representative teachers, 4Ps coordinators and parents per school and 1 school or division nurse per province (Table 1).

**Table 1:** Demographic characteristics of the participants for the qualitative study

	Group 1		Group 2	Group 3		Overall
	Parent (*n*=12)	Guardian (*n*=1)	Teacher (*n*=11)	4Ps coordinator (*n*=6)	School nurse (*n*=3)	*n*= 33
Gender						
Male	2	0	1	1	0	4
Female	10	1	10	5	3	29
Age, years	38	20	39	40.5	49	38
[min, max]	[27, 49]		[29, 64]	[28, 57]	[29, 53]	[20, 64]
Educational attainment						
Elementary level	1					1
Highschool level	6					6
College level	5	1	3	4		13
Master’s level			8	2	3	13
Province						
Agusan del Sur	5		1	1	2	10
Agusan del Norte	6	1	4	3	0	16
Surigao del Norte	1		6	2	1	7
Occupation						
None	9	1				10
Part time	2					2
Full time	1		11	6	3	21

4Ps= Pantawid Pamilyang Pilipino Program

### Data collection and analysis

Synchronous online FGDs were conducted via Google Meet (https://meet.google.com), after obtaining written informed consent. Participants were asked for permission to record the FGD, which was subsequently recorded for data analysis. Teacher participants also volunteered to facilitate parents who lacked internet access by inviting them to their offices, thereby enabling them to access the internet and participate in the focus group discussions. To minimize group bias effect, focused-group discussions were done separately with different stakeholders; teachers, 4Ps coordinators/school nurses, and parents who were 4Ps beneficiaries. Each group discussion comprised of 4 to 7 participants with the duration ranging from 60 to 90 min. Data collection and data analysis were employed in an iterative approach by two independent researchers (DH, MH, DA, and SB) until data saturation was observed.

Data obtained from the FGDs were first transcribed in Bisaya/Cebuano dialect and then translated into English for analysis. Codes and themes were independently identified and developed by two trained researchers (authors) using an iterative process. A constructivist modified-grounded theory analytical approach was used to identify emerging themes founded in the data [35, 36]. NVIVO 12 Pro software was used for the qualitative analysis and conducted in multiple phases and on four levels; [1] Translated quotes (open/initial coding) from the participants, [2] development of themes from the gathered codes, [3] focused and selective coding by group (see Supplementary Appendix 3, Additional files 2), and [4] development of conceptual framework from the grounded theory analysis.

## Results

### Sample characteristics of school-aged children

A total of 5541 secondary quantitative data on children and adolescents were obtained from 10 representative primary and secondary schools across Caraga Region. Of these, 436 (7.86%) were excluded from the study, resulting in the inclusion of 5107 (92.13%) children and adolescents. The excluded individuals fell into two categories: [1] non-residents of Caraga Region, and [2] children aged above 19 at the time of covariates and outcome assessment. Of the 5107 children and adolescents, 1812 (40.32%) were identified as members of 4Ps, while 3098 (60.68%) were non-4Ps beneficiaries (Table 2). The majority of both 4Ps and non-4Ps children belonged to non-indigenous groups (73.1%) and practiced Christianity as their religion (89.9%).

**Table 2:** Overall characteristics of school-aged children distributed by inclusion to 4Ps

Characteristics	4Ps ***N***= 1812	Non-4Ps ***N***=3098	Overall ***N***= 5107	***P*** value
**Sex of the child**				
Male	893 (49.3)	1553 (50.1)	2537 (49.7)	0.93
Female	896(49.4)	1525 (49.2)	2526 (49.5)	
Data missing	23 (1.3)	20 (0.6)	43 (0.9)	
**Age, category**				
Below 5	25 (1.4)	97 (3.1)	128 (2.5)	<0.001^a^
Between 6 and 10	810 (44.7)	2356 (76.0)	3207 (62.08)	
Between 11 and 19	968 (53.4)	602 (19.4)	1719 (33.7)	
Data missing	3 (0.2)	17 (0.5)	20 (0.4)	
**Educational level**				
Primary level	1188 (65.6)	2592 (83.7)	3833 (75.1)	<0.001^a^
Secondary level	624 (34.4)	495 (16.0)	1262 (24.7)	
Data missing	1	11 (0.4)	12 (0.2)	
**Ethnic group**				
Major tribes	262 (14.5)	211 (6.8)	474 (9.3)	<0.001^a^
Minor tribes	366 (20.2)	518 (16.7)	900 (17.6)	
Non-indigenous	1184 (65.3)	2368 (76.4)	3732 (73.1)	
Data missing	0	1	1	
**Religion**				
Christianity	1678 (92.7)	2714 (87.6)	4589 (89.9)	0.90
Islam	23 (1.3)	41 (1.3)	64 (1.3)	
others	8 (0.4)	19 (0.6)	27 (0.5)	
Data missing	101 (5.6)	324 (10.5)	425 (8.3)	
**Province**				
Agusan del Norte	1164 (64.2)	2487 (80.3)	3654 (71.6)	<0.001^a^
Agusan del Sur	185 (10.2)	239 (7.7)	425 (8.3)	
Surigao del Norte	45 (2.5)	209 (6.7)	445 (8.7)	
Surigao del Sur	418 (23.1)	140 (4.5)	559 (11.0)	
Data missing	1 (0.01)	23 (0.7)	24 (0.5)	

n(%)= count (proportion); 4Ps= Pantawid Pamilyang Pilipino Program; ^a^statistically significant

### Teachers’ and parents’ experiences and perception on 4Ps implementation

**Fig. 1:**
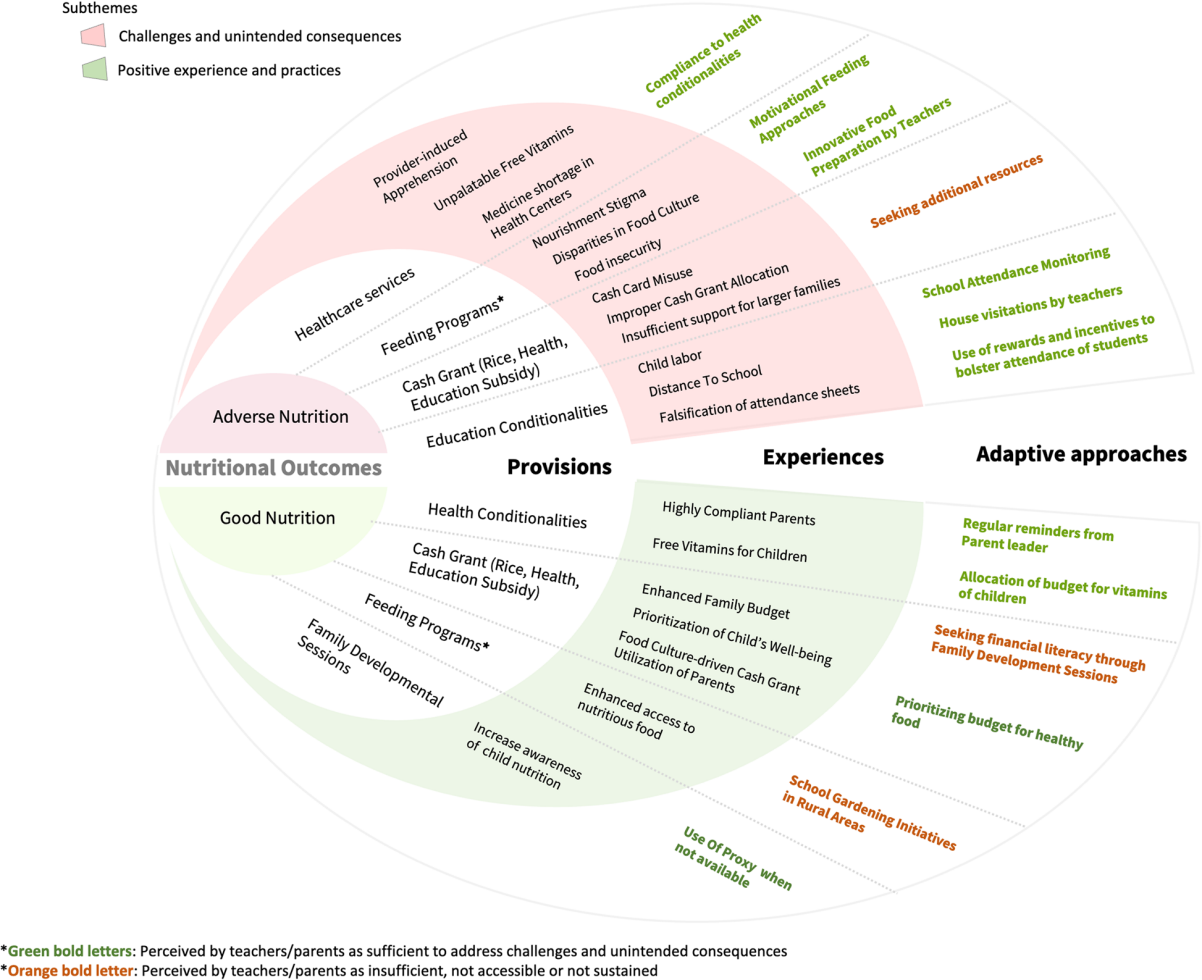
Conceptual framework on the influence of 4Ps on malnutrition in children and adolescents based on stakeholders’ experiences and perceptions

The analysis of the qualitative data revealed four interrelated themes that provided in-depth insights on the influence of the 4Ps on child nutrition based on 4Ps parent beneficiaries, teachers, 4Ps school coordinators, and school nurses. More precisely, these included: nutritional outcomes of 4Ps children and adolescents, provisions for enhanced child nutrition, experiences of parents and teachers in 4Ps implementation, and adaptive approaches for enhanced child nutrition and adherence to conditionalities (as shown in Fig. 1).

### Triangulation of quantitative and qualitative findings

#### Nutritional outcomes of 4Ps children and adolescents

**Fig. 2:**
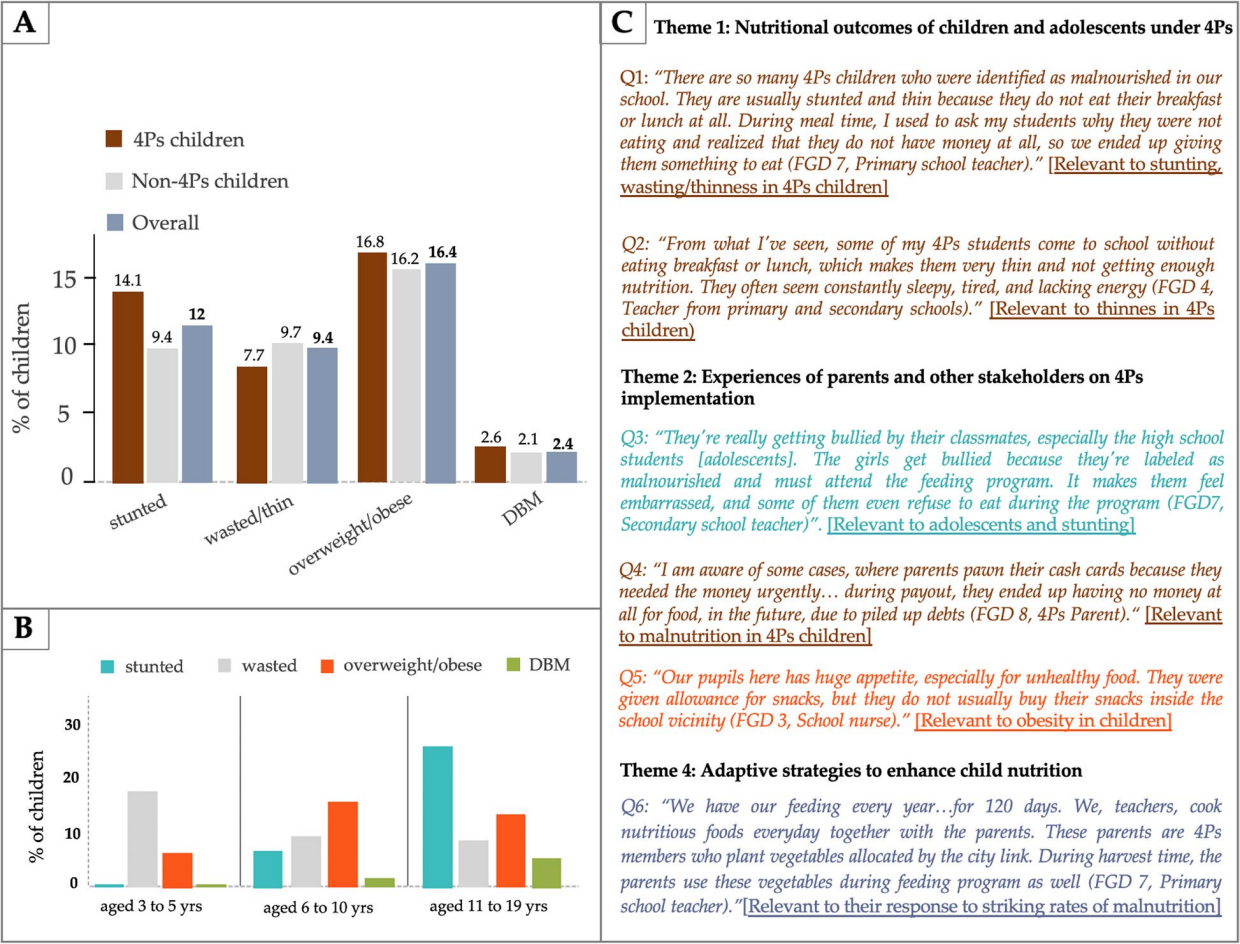
Joint display visual with (**A**) bar plots group by adverse nutritional outcomes among 4Ps and non-4Ps, (**B**) bar plots stratified by age group and by type of malnutrition outcome, and (**C**) representative quotations extracted from focus group discussion fieldnotes. Note: This display is based on the data from teachers, 4Ps coordinators, school nurses and 4Ps parent participants. The color of each bar correspond with the findings from both quantitative and qualitative analysis

The quantitative findings revealed striking rates of stunting (12.0%), wasting/thinness (9.4%), and overweight/obesity (16.4%) in children and adolescents (Fig. 2A). On the other hand, DBM was only prevalent in age-specific group, specifically among adolescents aged 11 to 19 (5.8%). 4Ps children and adolescents demonstrated higher rates of stunting overweight/obesity and DBM compared with non-4Ps (Fig. 2B).

The qualitative findings provided further support for these results based on the shared insights by teachers, indicating that most of the students who were identified as stunted or underweight, were also beneficiaries of 4Ps. A teacher, acting as a 4Ps coordinator in a primary school, noted a high proportion of stunted and wasted/thin students among the 4Ps beneficiaries in their school (Fig. 2C). The teachers also highlighted issues such as 4Ps students in secondary schools arriving at school without having eaten breakfast or lunch, resulting in persistent fatigue and drowsiness (Fig. 2C). Teachers collectively shared similar observations regarding 4Ps pupils in primary schools.

**Fig. 3:**
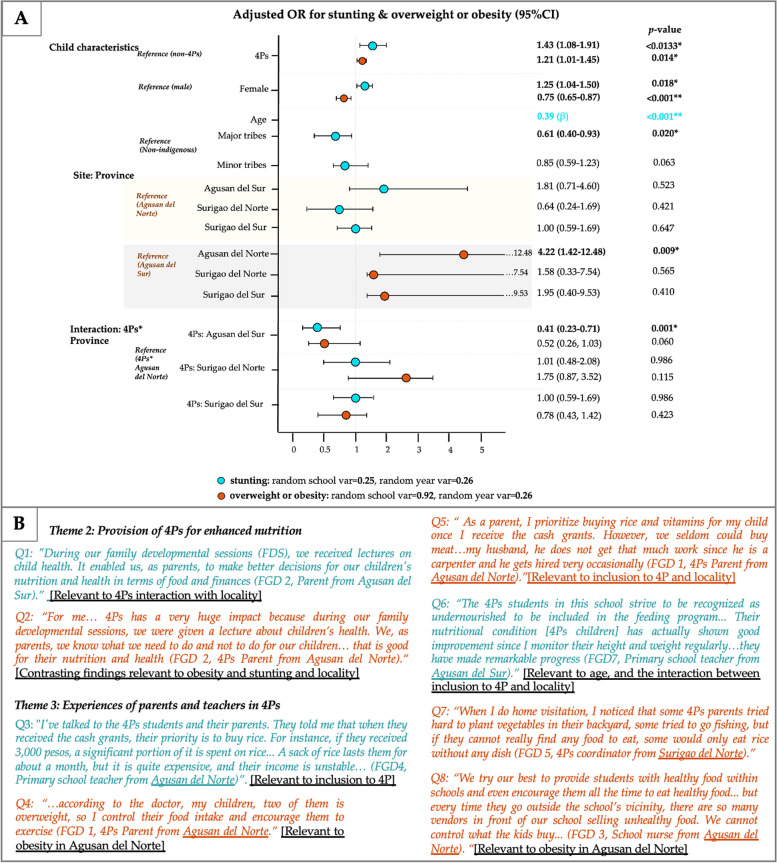
Joint display visual with (**A**) forest plot for stunting and overweight/obesity in children using random effects regression and (**B**) representative quotations extracted from focus group discussion fieldnotes relevant to stunting and overweight/obesity in children and adolescents. *significant p-value, var = variation, OR = odds ratios, 4Ps = *Pantawid Pamilyang Pilipino Program;* Adjusted OR = adjusted for age, sex, ethnicity, and province

**Fig. 4:**
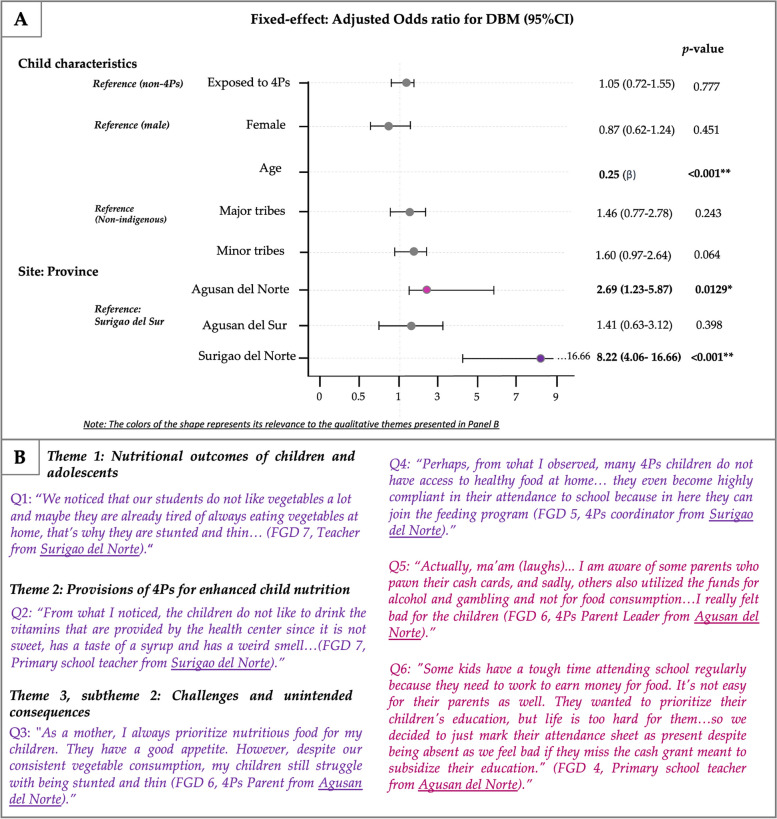
Joint display visual with (**A**) forest plot for stunting and overweight/obesity in children using random effects regression and (**B**) representative quotations extracted from focus group discussion fieldnotes relevant to DBM in 4Ps children and adolescents. *significant p-value, var = variation, OR = odds ratios; DBM = double burden of malnutrition; 4Ps = *Pantawid Pamilyang Pilipino Program;* Adjusted OR = adjusted for age, sex, ethnicity, and province

In terms of age group, quantitative findings showed a notable prevalence of stunting among adolescents aged 11 to 19 years (26.5%), and overweight and obesity being more prevalent in children aged 6 to 10 years (18.4%) (Fig. 2B). Similarly, the adjusted model for stunting (β = 0.39, *p* < 0.001) and DBM (β = 0.25, *p* < 0.001) revealed a significant association with age, after adjusting for confounding (Figs. 3A and 4A). However, age do not have significant association with overweight or obesity, after adjusting for inclusion to 4Ps, gender, ethnicity and locality.

In terms of gender, female children and adolescents exhibited a higher likelihood of stunting compared to their male counterparts (AOR = 1.25; 95%CI:0.1.04–1.50). In contrast, the likelihood of being overweight or obese were lower among females (AOR = 0.75; 95%CI: 0.65–0.87) compared with their counterparts, respectively (Fig. 3A).

Qualitative findings further reinforced these quantitative findings as the reasons behind these phenomena were complementary to the teachers’ observations regarding the attitudes of children and adolescents in schools. Teachers expressed concerns about adolescents, specifically female students in secondary schools, losing confidence to receive necessary provisions due to social factors like bullying, impacting nutrition improvement efforts (Fig. 2B). Conversely, the teachers noted that non-participating pupil in primary schools sought access to feeding program and would wait outside the program’s premises after its conclusion (Fig. 3B). However, some of the teachers and school nurses confined to the fact that these younger children also had high appetite and preference for unhealthy food, which was consistent with the quantitative findings regarding overweight/obesity rates in children aged 6 to 10 years.

#### Provisions of 4Ps for enhanced child nutrition

Our qualitative findings captured detailed description of the programmatic components and its provisions according to the insights shared by participating stakeholders. Parents and teachers highlighted the program’s ability to provide essential services and support to children. While certain health services were available to children outside the 4Ps, participation in the program ensured comprehensive monitoring and access to these nutritional and health services. These included regular deworming to address parasitic infections, regular health check-ups for children, engagement in feeding programs, and the provision of free vitamins for children, which were either obtained from the health centers or given during house-to-house visitation by health care workers (Fig. 1). However, what sets the 4Ps apart was the unique provision of a cash grant, contingent upon compliance with health and education conditions and attendance to family development sessions. Compliance to cash grant conditionalities provided the mechanism to sustain 4Ps intervention while school attendance monitoring attendance and regular health check-ups allowed parents to be observant in the cash grant conditionalities.

While 4Ps beneficiaries have access and were given prioritizations to health care services, these benefits were not fully appreciated by most of the 4Ps parents and teachers (Fig. 1, Table S6 & S7). Some parents from rural areas reported that free medicines and vitamins were undervalued. Additionally, the taste and odor of the provided vitamins and medicines are disliked by children, leading to a perception of burden rather than help (Fig. 4B). Parents of children living in remote islands also faced difficulties in accessing medicine and vitamins, as they must travel long distances for health consultations and medication purchases (Fig. 1, Table S7).

On the other hand, all parent participants reported high compliance with the health conditionalities of the program and attendance to family developmental sessions. Several parents perceived family developmental sessions as an opportunity to learn about child health as well as financial management (Fig. 3B). These sessions empowered parents to make informed decisions and take proactive steps to enhance their children’s well-being. Furthermore, the majority of 4Ps parents expressed gratitude for the provision of cash grants to support their child’s nutrition and educational, emphasizing its importance in subsidizing their budget for their child’s food, education, and health.

#### Experiences of parents and teachers

The diverse experiences shared by parents and teachers regarding the 4Ps were presented into two following subthemes: (1) challenges and unintended consequence of 4Ps implementation, and (2) positive experiences and practices of parents and teachers regarding 4Ps implementation.

#### Subtheme 1: challenges and unintended consequences of 4Ps implementation

Results from mixed-effect logistic regression showed that the odds of stunting as well as overweight/obesity among 4Ps beneficiaries were higher compared with non-4Ps child, after adjusting for gender, age, ethnicity, and locality (AOR = 1.43, 95%CI: 1.08–1.91, and AOR = 1.21, 95%CI: 1.01–1.45, respectively) (Fig. 3A). Nonetheless, inclusion to 4Ps showed no significant association with DBM (AOR = 1.05, 95%CI 0.72–1.55) (Fig. 4A).

The qualitative findings further supported the quantitative findings, emphasizing the complex challenges and unintended consequences experienced by 4Ps child beneficiaries. These challenges encompassed a wide range of factors, including difficulties related to distance to school, child labor, illness, food insecurity, limited support for larger families, nourishment stigma, cultural disparities, provider concerns, unpalatable free vitamins, misuse of cash cards, and inappropriate allocation of cash grants (Fig. 1). One of the teachers shared that parents prioritized the purchase of rice once cash grants were received. However, due to the high cost of rice, large family size and unstable livelihood, parents still continued to experience extreme difficulties in meeting their basic needs, which was also confirmed by one of the 4Ps parents (Fig. 3B).

Unintended consequences were also identified such as inappropriate cash grant allocation and the use of cash cards, which had diversified from a mode of cash release to something as a guaranty for loan with or without interest (Fig. 4B). Despite parents’ awareness about the penalties imposed by the 4Ps implementers against cash card pawning, some continued to practice these illegal acts due to extremely difficult situations and urgent needs. These included urgent needs of the child and for food consumption with no other means or knowledge on how and where to access lending institutions or associations nor earn money to buy their needs while waiting for the cash grant (Table S7). Another unintended consequences observed was the falsification of school attendance by teachers as a means to ensure 4Ps students received their cash grants. Teachers expressed their concerns about the difficulties faced by 4Ps students, particularly those who engaged in part-time work to support their families. To ensure these students still benefited from the program, some teachers marked their attendance as present and conducted home visits (Fig. 4B).

When we examined the interaction between inclusion to 4Ps and geographical location, 4Ps children in Agusan del Norte had approximately four times higher odds of obesity compared with those in Agusan del Sur (AOR = 4.22, 95%CI: 1.42–12.48). The qualitative findings further unveiled insights regarding the presence of an obesogenic environment in urban areas of Agusan del Norte. This exposure to urbanized settings were identified by school nurses and teachers in primary schools to be linked to the availability and accessibility of unhealthy or obesogenic food. Specifically, teachers and school nurses emphasized their efforts to provide healthy food within the school premises, but expressed concerns about the presence of food vendors selling unhealthy options just outside the school’s vicinity (Figs. 2B and 3B).

On another note, children in the province of Surigao del Norte were 8.22 times more likely to have DBM compared to children in the province of Surigao del Sur (AOR = 8.22; 95%CI: 4.06–16.66) (Fig. 3A). The qualitative findings corroborated with these results, as teachers in Surigao del Norte also noted a general disinterest among children in consuming vegetables, speculating on its potential contribution to their concurrent stunting and thinness (Fig. 4B). However, despite parents’ conscientious efforts to provide nutritious meals with regular vegetable intake, their children continued to exhibit signs of concurrent stunting and thinness.

#### Subtheme 2: positive experiences and practices

In terms of the positive association of 4Ps with specific geographical location, our quantitative findings demonstrated a lower likelihood of stunting and overweight/obesity among 4Ps children who were living in Agusan del Sur (AOR = 0.41, 95%CI: 0.23–0.71; AOR = 0.52, 95%CI: 0.26–1.03, respectively) compared with Agusan del Norte.

The qualitative findings complemented these results by uncovering positive experiences and practices reported by teachers and parents. These included high compliance of 4Ps parents, acquisition of free vitamins for children, enhanced family budget for food, health, and education, and active participation to family developmental sessions and feeding programs, particularly in Agusan del Sur (Fig. 3B, Table S7). More precisely, one of the 4Ps parents from Agusan del Sur attested their awareness of child nutrition through their attendance at family developmental sessions and feeding programs. This awareness empowered them to make informed decisions and further promote healthy food culture within their families. However, it is noteworthy to acknowledge that certain parents from Agusan del Norte also divulged their commendable approaches, including the prioritization of purchasing rice and vitamins for their children upon receiving the cash grant, as well as adopting healthy practices to address their children’s overweight condition. They also emphasized the positive impact of cash grant on enhanced affordability for nutritious food and vitamins for their children since their inclusion to 4Ps (Fig. 1, Table S7).

The food culture at home and in the many communities were believed to influence child’s nutrition, which varies with family sizes and access to healthy yet affordable food. Though cash grant allowed parent to reward children to eat as much and according to what they know is nutritious, the food culture on nutritious and healthy diet comprised of vegetables, fruits and meat is preserved and repeatedly promoted despite child’s preference (Fig. 4B, Table S7). Furthermore, one of the 4Ps coordinator from Surigao del Norte highlighted the interesting behavior of highly compliant 4Ps children in education, especially those who do not have sufficient access to health food at home. Specifically, she reported high attendance among 4Ps children in primary schools as these children saw this as an opportunity to join feeding programs.

#### Theme 4: Adaptive approaches to enhance child nutrition

The qualitative findings highlighted the adaptive approaches employed by teachers and parents to enhance the nutrition of children and adolescents. Parents enrolled in the 4Ps demonstrated compliance with health conditionalities, actively sought additional food resources, diligently followed parent leader’s reminders, allocated a specific budget for children’s vitamins and healthy food, actively participated in family developmental sessions, and involved other family members in attending these sessions (Fig. 1, Table S6).

Teachers employed strategies by closely monitoring of 4Ps students’ attendance, conducting home visits for frequently absent students, using incentives to encourage attendance, initiating school gardening in rural areas, and employing innovative food preparation techniques during feeding programs (Table S5 & S7). One of the teachers particularly shared her strategic approach to bolstering students’ school attendance:

*“We always encourage them to come to school to study, so we use rewards and incentives to motivate them. As a result, the children started to develop a desire to go to school because of the rewards they received (Teachers in rural school; FGD 4, Primary Teachers).“*.

During feeding programs, teachers recognized the food preferences of their students, particularly their aversion to vegetables. To address this, teachers shared that they innovated and modified the food they prepared, making it tastier and more appealing to children than their previous feeding programs. They also employed motivational feeding approaches to encourage adolescents who might have felt embarrassed to attend the feeding program, emphasizing the significance of consuming nutritious food to combat malnutrition. Specifically, teachers in Agusan del Sur reported their strategy related to the conduct of feeding programs within their schools such as establishment of vegetable gardens by 4Ps mothers (Fig. 2B). Unfortunately, one of the 4Ps coordinator shared that this initiative was not sustained due to insufficient budget for building a proper nursery.

Several parents also expressed their willingness to remain compliant to health conditionalities amidst the challenges faced in accessing health services in their communities. They vividly described how health workers diligently conducted house-to-house visits, administering deworming pills and reminding parents about the importance of regular check-ups, especially for pregnant women and young children (Table S7). Few parents also reported that to enhance their family budget, they planned to develop their financial literacy skills, which focused on child nutrition and responsible cash grant utilization. However, this remained insufficient for many of the parents due to other difficulties, especially those who have large families (Fig. 1).

## Discussions

This concurrent mixed-method study provided valuable insights on the association between inclusion to 4Ps on child malnutrition and how the programmatic components had influenced such outcomes based on parents’ and teachers’ perceptions and experiences.

### Malnutrition in children and adolescents

Our quantitative findings revealed high rates of stunting, wasting/thinness, overweight/obesity, and age-specific double burden of malnutrition (DBM) among children and adolescents. Similarly, a recent school-based study conducted in the Philippines reported striking rates of stunting and DBM among adolescents, with an estimated prevalence of 20.3% and 2.4%, respectively, based on a sample size of 3488 participants [37].

Our findings further indicated that older age was significantly associated with a higher likelihood of stunting and DBM. A cross-sectional study conducted among children aged 6 to 12 in rural community of Ethiopia also showed higher odds of stunting among children aged 10 to 12 compared with their younger counterparts [38]. Interestingly, a study conducted in Peru examined the age of exposure to the Juntos Conditional Cash Transfer program and reported a significant reduction in stunting among children exposed to the program at the age of 3 and below [39]. However, their findings reported no significant effect on the nutritional status for children exposed to the program at the age of 5 to 7. Nonetheless, these findings suggested the importance of targeting the appropriate age for inclusion in Conditional Cash Transfer programs to effectively address malnutrition in children and adolescents.

While we observed a potential link between older age and a higher likelihood of overweight/obesity, the findings did not attain statistical significance in establishing this association. Nonetheless, a recent report from the World Bank suggested an increasing trend of overweight/obesity rates among Filipino children since the latest survey in 2018, with highest increase observed among older children (aged 10 to 19 years old) [40]. Evidence from a systematic review and meta-analysis of over 200,777 participants reported that obese children and adolescents are five times more likely to be obese in adulthood, with around 55% of obese children becoming obese in adolescence and 80% of obese adolescents remaining obese into adulthood. The findings indicated that as children grow older, their likelihood of overweight/obesity in adolescence and adulthood increases significantly [41]. This underscores the importance of addressing these adverse nutritional outcome at an early age to prevent long-term consequences and highlights the need for targeted nutritional interventions in older age groups to combat the persistent burden of overweight/obesity.

### Insights on the influence of 4Ps on malnutrition in children and adolescents

Results from the mixed-effect logistic regression showed that the odds of stunting and overweight/obesity were higher among 4Ps beneficiaries compared with non-4Ps child. Moreover, the proportion of concurrent stunting and wasting/thinness or overweight/obesity, was slightly higher among 4Ps child beneficiaries (2.6%) than their non-4Ps counterparts (2.1%). In the Philippines, although CCT program showed a great potential to improve nutritional outcomes of children living in poverty, the World Bank reported a concerning trend regarding the current impact of the 4Ps on improving these outcomes among Filipino children [9]. The report indicated a decline in the program’s influence over time. Additionally, a significant disparity in stunting rates between high- and low-income households was observed in the Philippines, with children from the poorest families being more than twice as likely to experience stunting compared to those from wealthier families [9, 42]. Furthermore, recent studies conducted in Colombia, Mexico, and Peru reported the potential dual effects of CCT programs, indicating a potential decrease in stunting rates but an increase in the risk of overweight/obesity among children in the program [16, 43, 44]. However, findings from a systematic review reported to have insufficient evidence on the impact of CCT on overweight or obesity in children to make a firm conclusion due to very limited studies on this topic [45].

Our qualitative findings further reinforced this evidence based on the challenges shared by teachers and 4Ps parents in improving children’s nutrition and the unintended consequences of the 4Ps. Specifically, teachers expressed deep concerns about the insufficiency of cash grants provided to families, particularly those with a large number of children and unstable family income. In line with our qualitative findings, a randomized trial in Mexico showed that increasing cash grants in a CCT program improved nutritional outcomes of children, including lower stunting and overweight prevalence and higher height-for-age Z-scores and hemoglobin concentrations [46]. Nonetheless, our qualitative findings showed that cash grant release has been claimed by parents to be very helpful in the nutrition of their children and entire family. Beneficiaries affirmed that the cash grant release changed the impoverished condition of their family to enjoy lavishing in eating delicious food and engaging recreation together. However, this 4Ps benefit in several beneficiaries did not guarantee sustained provision due to the meager budget and low family income.

On another note, our findings brought forth the challenges shared by 4Ps parents regarding their relentless struggles in enhancing their children’s nutritional status despite regular vegetable intake. A study on the influence of dietary patterns among children reported an intriguing observation, indicating that children who adhered to a carbohydrate-protein rich dietary pattern exhibited a lower likelihood of experiencing stunting compared to those who consumed diets with low-intake of these essential nutrients [47]. This suggested that focusing solely on a single food group, such as vegetables, may not be sufficient to address the multifaceted issue of child malnutrition. A comprehensive approach that considered the overall dietary pattern, including adequate intake of carbohydrates and proteins and higher adherence to these dietary patterns, may be necessary to effectively improve children’s nutritional status and reduce the risk of adverse nutritional outcomes [47, 48].

### Influence of geographical variations on the link between 4Ps and malnutrition

Our study also explored the interplay between 4Ps, geographical location, and malnutrition in children and adolescents. A closer examination on the influence of geographical location demonstrated that 4Ps children and adolescents in Agusan del Sur (representing predominantly rural areas) exhibited a lower likelihood of stunting compared to those in Agusan del Norte (characterized by predominantly urban settings). Conversely, children and adolescents in Agusan del Norte demonstrated a four-fold higher likelihood of overweight/obesity compared to those in Agusan del Sur. Similarly, reports from the World Bank highlighted the variations in malnutrition rates across different regions in the Philippines [10]. A study conducted in Cabanatuan, Philippines, further reported high variations of malnutrition density across different areas in the city, and emphasized the importance of refocusing nutritional programs in areas with high malnutrition density per population [49]. In light of these evidence, our study highlighted the substantial influence of geographical factors on malnutrition outcomes as well as the impact of 4Ps on these outcomes.

Our qualitative findings provided further insights on the adaptive strategies that were employed by teachers in schools to address the challenges on child malnutrition, including the preparation of tasty yet health food during feeding programs and establishment of school nurseries for 4P mothers. On the other hand, challenges posed by the obesogenic environment in urban schools in Agusan del Norte, Philippines remained to be the main issue reported by school nurses and teachers. They expressed dedication to providing nutritious meals on-campus but expressed concern about the availability of unhealthy food options from vendors located outside school premises. Nonetheless, attendance of parents to family developmental session (FDS) had increased their knowledge on ensuring their children’s nutrition. Moreover, teachers, parents, and school coordinators made joint efforts in the conduct of feeding program to train children on healthy food and provide starving children through results of health monitoring. Other children were noted to be intimated with wanting nutrition but other starving children considered this as opportunity to be included in feeding programs.

Numerous studies have also consistently indicated a strong link between urban settings and heightened susceptibility to overweight and obesity, attributed to the obesogenic environment prevalent in such areas [35, 40, 50]. In the Philippines, the likelihood of overweight/obesity among Filipino adolescents living in urban areas were 34% higher than their counterparts in rural areas [40]. A study conducted in Indonesia also demonstrated a strong association between an obesogenic environment in the community and nearby schools, highlighting its detrimental impact on children’s nutritional outcomes [35]. These findings emphasized the crucial role of tackling the detrimental effects of obesogenic urban environments to effectively enhance child nutrition in the context of 4Ps implementation, particularly in combating the rising rates of overweight and obesity.

### Strength and limitations of the study

The strength of our study lies in the utilization of a concurrent mixed-method study design, which allowed for the simultaneous triangulation of both quantitative and qualitative data. This approach enabled us to define more accurately the relationship between the conditional cash transfer program (CCT) and child nutritional status. The repeated cross-sectional design used during the quantitative study ensured a representative sample of study participants, which allowed us to produce unbiased estimates on the association between inclusion to 4Ps and malnutrition outcomes in children. Moreover, we employed a robust methodological approach by using school and year random-effect specifications and adjusting for unobserved differences between schools and underlying trends. In parallel with the quantitative study, the qualitative study enabled us to understand the stakeholders’ experiences on the implementation of child nutrition intervention programs and other important factors that contributed to stunting, overweight/obesity, and double burden of malnutrition (DBM) in a more contextualized approach.

Our study has several limitations. We were unable to acquire data regarding the duration of children’s exposure to the 4Ps. This data would have facilitated an exploration of potential effect modifications of the program on child malnutrition. Additionally, influential variables such as inclusion in feeding and deworming programs, family size, and socio-economic variables such as quintiles of wealth index and mother’s education were not available in school forms provided during the analysis of the quantitative study. Nevertheless, these influential variables were evident in the qualitative findings, which comprehensively captured the experience of parents, teachers and other stakeholders with regards to other nutritional intervention, both within the school environment and across homes and communities.

## Conclusion

Our findings indicate that inclusion to 4Ps was associated with higher likelihood of stunting and overweight/obesity among children and adolescents. Moreover, the program’s association with malnutrition was significantly influenced by geographic variations, with lower likelihood of stunting observed in rural areas compared with urban. Qualitative findings further provided valuable insights on the complex challenges and unintended consequences faced by beneficiaries in different provinces, including food insecurity, presence of obesogenic environment in urban areas, insufficient cash grant for large families, nourishment stigma among adolescents, cash card misuse, and inappropriate cash grant allocation. On the other hand, parents and teachers have shared their experiences of developing adaptive approaches to encourage compliance with the program’s education conditionalities and enhance child nutrition, both within the school environment and across homes and communities. Nonetheless, it is crucial to prioritize the development and implementation of tailored support strategies for child nutrition, depending on the needs of the community. These strategies should aim not only to mitigate malnutrition outcomes among children and adolescents but also to proactively address the challenges and unintended consequences faced by both the beneficiaries and other relevant stakeholders. Furthermore, a more comprehensive evaluation of CCT programs’ long-term impact on malnutrition in children is needed to thoroughly understand their effectiveness, identify potential limitations, and devise strategies for sustainable and substantial improvement in child nutrition outcomes.

### Supplementary Information

**Additional file 1: Appendix 1.** A priori assumption using direct acyclic graph (DAG) for the link between 4Ps inclusion and malnutrition in children and adolescents. **Figure S1.** Direct acyclic graph on the causal relationship between conditional cash transfers and child nutritional status. **Appendix 2. **Results from the quantitative analyses.**Table S1. **Nutritional status of school children and year of measurement by age group. **Table S2. **Mixed effects logistic for stunting among children aged between 3 and 19 in Caraga Region. **Table S3. **Mixed effects logistic for overweight/obesity among children aged between 3 and 19 in Caraga Region. **Table S4. **Mixed effects logistic for DBM (concurrent stunting and wasting/thinness or overweight/obesity) in children and adolescents.

**Additional file 2: Appendix 3.** Open codes, focused codes and clusters developed based on the data from the focused group discussions of parents, teachers, 4Ps coordinators and school nurses. **Table S5. **Sample FGD obtained from teacher participants using open codes, focused codes, and clusters.** Table S6. **FGD-sample obtained from teacher participants. **Table S7. **FGD-sample obtained from parent participants under 4Ps.

## Data Availability

The dataset used for the quantitative study are made available from the DepEd school divisions and representative school. Thus, administrative permissions are required to access the raw data from this organization. Public access to the database is open upon permission. On the other hand, deidentified data on qualitative study is available upon request to the corresponding author- Deborah Jael Herrera via email: djherrera5947@gmail.com.
