# Supplementary material for: Mixed-method study on the association between inclusion to conditional cash transfer program and the multiple faces of malnutrition in children and adolescents aged 3 to 19 years: a school-based evidence from Caraga Region, the Philippines
Source: BMC Pediatr. 2023 Dec 13;23:630. doi: 10.1186/s12887-023-04438-8 (PMC10717276; doi:10.1186/s12887-023-04438-8)
Supplement: Supplementary file 1 — Additional file 1: Appendix 1. A priori assumption using direct acyclic graph (DAG) for the link between 4Ps inclusion and malnutrition in children and adolescents. Figure S1. Direct acyclic graph on the causal relationship between conditional cash transfers and child nutritional status. Appendix 2. Results from the quantitative analyses.Table S1. Nutritional status of school children and year of measurement by age group. Table S2. Mixed effects logistic for stunting among children aged between 3 and 19 in Caraga Region. Table S3. Mixed effects logistic for overweight/obesity among children aged between 3 and 19 in Caraga Region. Table S4. Mixed effects logistic for DBM (concurrent stunting and wasting/thinness or overweight/obesity) in children and adolescents. [file 12887_2023_4438_MOESM1_ESM.docx]

**Supplementary material**

**Appendix 1.** A priori assumption using direct acyclic graph (DAG) for the link between 4Ps inclusion and malnutrition in children and adolescents.

The Direct Acyclic Graph (**Figure S1**) was mapped, using a web-browser software called *dagitty*, to determine a priori assumptions about the relationships between CCTs and child nutrition outcomes and to identify confounding variables that require conditioning during data analysis. The DAG-implied adjustment sets for estimating such causal effect are socio-cultural status (SCS), socio-demographic status (SDS), socio-economic status (SES). More precisely, SCS was assessed through data on participant’s ethnicity, religion, and their mother tongue, while data on SDS includes child’s sex and child’s age, child’s grade level, school division, attending school, rural vs urban area, and province. However, data on SES were not obtained since data shared and collected by the representative schools were limited.

**Figure S1.** Direct acyclic graph on the causal relationship between conditional cash transfers and child nutritional status


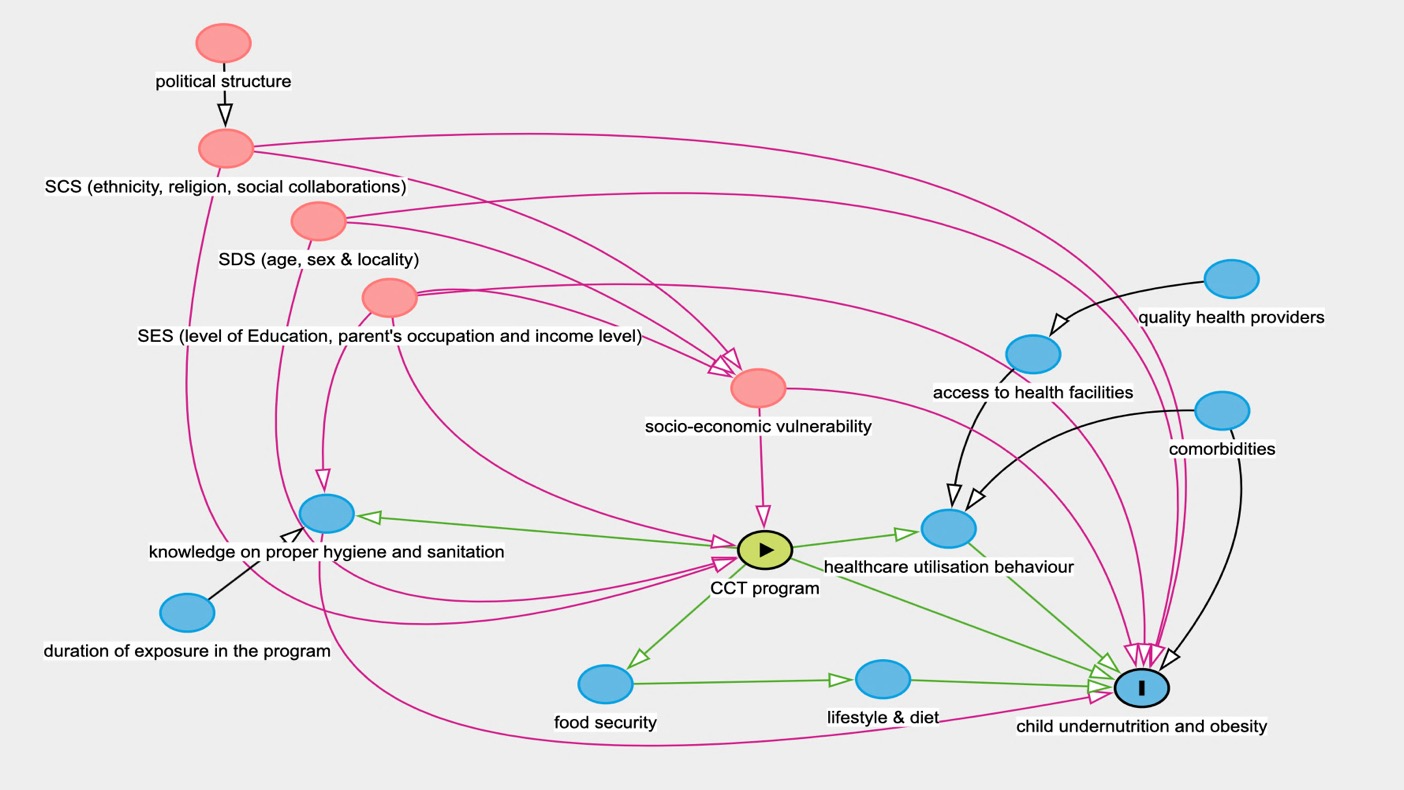


**Appendix 2. Results from the quantitative analyses**

**Table S1.** Nutritional status of school children and year of measurement by age group

|  | **Children aged 3 to 5 years** | **Children aged 6 to 10 years** | **Children aged 11 to 19 years** |
| --- | --- | --- | --- |
| **All children** | (N= 306) | (N=3277) | (N=1524) |
| Age, years | 4.60 (0.46)^a^ | 8.14 (1.72)^a^ | 13.4 (1.92)^a^ |
| **Nutritional status** § |  |  |  |
| Stunting | 0 | 209 (6.4%) | 404 (26.5%) |
| Wasting | 61 (19.9%) | 310 (9.5%) | 107 (7.0%) |
| Overweight or obesity | 19 (6.2%) | 604 (18.4%) | 216 (14.2%) |
| Concurrent stunting & wasting,  overweigh or obesity | 0 | 52 (1.6%) | 89 (5.8%) |
| Weight, kg | 17.0 (3.50) ^a^ | 26.4 (8.65) ^a^ | 41.0 (9.21) ^a^ |
| Height, m | 1.09 (0.07) ^a^ | 1.27 (0.12) ^a^ | 1.47 (0.09) ^a^ |
| BMI, kg/m^2^ | 14.2 (2.02) ^a^ | 16.0 (3.00) ^a^ | 18.7 (3.00) ^a^ |
| Weight-for-age z* (WAZ) | -0.299 (1.42) ^a^ | -0.196 (1.51) ^a^ | NA |
| Height-for-age z* (HAZ) | 0.517 (1.64) ^a^ | -0.085 (1.37) ^a^ | -1.25 (1.71) ^a^ |
| BMI-for-age z* (BAZ) | -0.946 (1.50) ^a^ | -0.251 (1.52) ^a^ | -0.235 (1.25) ^a^ |
| **Year of measurement** |  |  |  |
| 2016 | 0 | 2 | 67 |
| 2017 | 32 | 114 | 203 |
| 2018 | 49 | 335 | 394 |
| 2019 | 160 | 2605 | 583 |
| 2020 | 62 | 161 | 8 |
| 2021 | 0 | 14 | 256 |

^a^ Data are mean (SD). BMI= Body Mass Index. z= Z scores. *Standardize Weight-for-age, Height-for-age, and BMI-for-age scores calculated according to WHO Child Growth Standard for aged 5 to 19 years of age and 5 below. § Proportions of children with malnutrition were calculated by dividing the total number of cases (with stunting, wasting, overweight/obesity, or DBM) by the total number of 4Ps or non-4Ps children x 100

The standard deviation for school’s random intercept indicated that the odds of stunting among children in school varied around the average intercept by about 0.389 unit for model 1 and about 0.256 unit for model 2 (**Table S2**).

**Table S2.** Mixed effects logistic for stunting among children aged between 3 and 19 in Caraga Region.

|  | **Model 1_s_** | | | | **Model 2_s_** | | | |
| --- | --- | --- | --- | --- | --- | --- | --- | --- |
| **Parameter** | **Est.** | **SE** | ***p*** | **OR (CI)** | **Est.** | **SE** | ***p*** | **AOR (CI)** |
| **Fixed effects:** | | | | | | | | |
| **Intercept** | 5.875 | 0.414 | **<0.001*** |  | 6.107 | 0.471 | **<0.001**** |  |
| **Inclusion to 4Ps** |  |  |  |  |  |  |  |  |
| Non-4Ps |  |  |  | *reference* |  |  |  | *reference* |
| 4Ps | 0.207 | 0.103 | **0.044*** | 0.81 (0.66-0.99) | 0.360 | 0.145 | **0.0133*** | **1.43 (1.08-1.91)** |
| **Sex (Female)** | -0.226 | 0.093 | **0.015*** | 0.80 (0.66-0.96) | -0.221 | 0.093 | **0.018*** | **1.25 (1.04-1.50)** |
| **Age** | 0.390 | 0.024 | **<0.001*** |  | 0.397 | 0.025 | **<0.001**** |  |
| **Ethnicity** |  |  |  |  |  |  |  |  |
| Major tribes |  |  |  |  | 0.491 | 0.212 | **0.020*** | **0.61 (0.40-0.93)** |
| Minor tribes |  |  |  |  | -0.332 | 0.179 | 0.063 | 0.85 (0.59-1.23) |
| Non-indigenous |  |  |  |  |  |  |  | *reference* |
| **Province** |  |  |  |  |  |  |  |  |
| Agusan del Norte |  |  |  |  |  |  |  | *reference* |
| Agusan del Sur |  |  |  |  | 0.306 | 0.480 | 0.523 | 1.81 (0.71-4.60) |
| Surigao del Norte |  |  |  |  | 0.445 | 0.554 | 0.421 | 0.64 (0.24-1.69) |
| Surigao del Sur |  |  |  |  | -0.206 | 0.450 | 0.647 | 1.00 (0.59-1.69) |
| **Interaction~ 4Ps*Province** |  |  |  |  |  |  |  |  |
| **4Ps: Agusan del Norte** |  |  |  |  |  |  |  | *reference* |
| 4Ps: Agusan del Sur |  |  |  |  | -0.898 | 0.285 | **0.001**** | **0.41 (0.23-0.71)** |
| 4Ps: Surigao del Norte |  |  |  |  | 0.006 | 0.371 | 0.986 | 1.01 (0.48-2.08) |
| 4Ps: Surigao del Sur |  |  |  |  | 0.001 | 0.267 | 0.995 | 1.00 (0.59-1.69) |
| **Random effects:** | | | | | | | | |
| **Groups**  **..** | | **Var** | **Std. dev** | **N group** | **Var** | **Std. dev** | **N group** | ***p^a^* Model 2** |
| **Year measured (intercept)**  (Intercept) | | 0.389 | 0.624 | 6 | 0.256 | 0.534 | 6 |  |
| **School (intercept)**  (Intercept) | | 0.239 | 0.489 | 10 | 0.252 | 0.502 | 10 |  |
| **AIC** | | 3184 |  |  | 3182 |  |  | **0.021*** |

*p=* p-value*, ^a^* ANOVA, Est= estimate, SE= standard error, OR= odds ratio, CI= confidence interval, Var= Variance, Std. dev= standard deviation,

**Table S3.** Mixed effects logistic for overweight/obesity among children aged between 3 and 19 in Caraga Region.

|  | **Model 1_s_** | | | | **Model 2_s_** | | | |
| --- | --- | --- | --- | --- | --- | --- | --- | --- |
| **Parameter** | **Est.** | **SE** | ***p*** | **OR (CI)** | **Est.** | **SE** | ***p*** | **AOR (CI)** |
| **Fixed effects:** | | | | | | | | |
| **Intercept** | -2.219 | 0.451 | **<0.001*** |  | -1.441 | 0.527 | **<0.006*** |  |
| **Inclusion to 4Ps** |  |  |  |  |  |  |  |  |
| Non-4Ps |  |  |  | *reference* |  |  |  | *reference* |
| 4Ps | 0.178 **Table S1.** Mixed effects logistic for stunting among children aged between 3 and 19 in Caraga Region.   \|  \| **Model 1_s_** \| \| \| \| **Model 2_s_** \| \| \| \| \| --- \| --- \| --- \| --- \| --- \| --- \| --- \| --- \| --- \| \| **Parameter** \| **Est.** \| **SE** \| ***p*** \| **OR (CI)** \| **Est.** \| **SE** \| ***p*** \| **AOR (CI)** \| \| **Fixed effects:** \| \| \| \| \| \| \| \| \| \| **Intercept** \| -6.082 \| 0.427 \| **<0.001*** \| 0.0001 \| -5.976 \| 0.490 \| **<0.001*** \| 0.0002 \| \| **Inclusion to 4Ps** \|  \|  \|  \|  \|  \|  \|  \|  \| \| Non-4Ps \|  \|  \|  \| *reference* \|  \|  \|  \| *reference* \| \| 4Ps \| 0.207 \| 0.103 \| **0.044*** \| 0.81 (0.66-0.96) \| 0.360 \| 0.145 \| **0.0133*** \| 0.70 (0.52, 0.93) \| \| **Sex (Female)** \| -0.226 \| 0.093 \| **0.015*** \| 0.80 (0.66-0.96) \| -0.221 \| 0.093 \| **0.018*** \| 0.80 (0.67-0.96) \| \| **Age** \| 0.390 \| 0.024 \| **<0.001*** \| 1.48(1.41-1.55) \| 0.397 \| 0.025 \| **<0.001*** \| 1.49 (1.41-1.56) \| \| **Ethnicity** \|  \|  \|  \|  \|  \|  \|  \|  \| \| Major tribes \|  \|  \|  \|  \| 0.491 \| 0.212 \| **0.020*** \| 1.63 (1.08-2.47) \| \| Minor tribes \|  \|  \|  \|  \| -0.332 \| 0.179 \| 0.063 \| 0.72 (0.50-1.01) \| \| Non-indigenous \|  \|  \|  \|  \|  \|  \|  \| *reference* \| \| **Province** \|  \|  \|  \|  \|  \|  \|  \|  \| \| Agusan del Norte \|  \|  \|  \|  \|  \|  \|  \| *reference* \| \| Agusan del Sur \|  \|  \|  \|  \| 0.306 \| 0.480 \| 0.523 \| 1.36 (0.53-3.48) \| \| Surigao del Norte \|  \|  \|  \|  \| 0.445 \| 0.554 \| 0.421 \| 1.56 (0.53-4.62) \| \| Surigao del Sur \|  \|  \|  \|  \| -0.206 \| 0.450 \| 0.647 \| 0.81 (0.34-1.96) \| \| **Interaction~ 4Ps*Province** \|  \|  \|  \|  \|  \|  \|  \|  \| \| **4Ps: Agusan del Norte** \|  \|  \|  \|  \|  \|  \|  \| *reference* \| \| 4Ps: Agusan del Sur \|  \|  \|  \|  \| -0.898 \| 0.285 \| **0.001*** \| 2.45 (1.40-4.30) \| \| 4Ps: Surigao del Norte \|  \|  \|  \|  \| 0.006 \| 0.371 \| 0.986 \| 0.99 (0.48-2.06) \| \| 4Ps: Surigao del Sur \|  \|  \|  \|  \| 0.001 \| 0.267 \| 0.995 \| 0.99 (0.59-1.69) \| \| **Random effects:** \| \| \| \| \| \| \| \| \| \| **Groups**  **..** \| \| **Var** \| **Std. dev** \| **N group** \| **Var** \| **Std. dev** \| **N group** \| ***p^a^* Model 2** \| \| **Year measured (intercept)**  (Intercept) \| \| 0.389 \| 0.624 \| 6 \| 0.256 \| 0.534 \| 6 \|  \| \| **School (intercept)**  (Intercept) \| \| 0.239 \| 0.489 \| 10 \| 0.252 \| 0.502 \| 10 \|  \| \| **AIC** \| \| 3184 \|  \|  \| 3182 \|  \|  \| **0.021*** \|   *p=* p-value*, ^a^* ANOVA, Est= estimate, SE= standard error, OR= odds ratio, CI= confidence interval, Var= Variance, Std. dev= standard deviation | 0.083 | **0.032*** | **1.19 (1.01-1.40)** | 0.121 | 0.087 | **0.0141*** | **1.24 (1.04-1.47)** |
| **Sex (Female)** | -0.287 | 0.434 | **0.000***** | **0.75 (0.65-0.87)** | -0.289 | 0.075 | **0.000***** | **0.75 (0.65-0.87)** |
| **Age** | 0.390 | 0.024 | **<0.010*** |  |  |  |  |  |
| **Province** |  |  |  |  |  |  |  |  |
| Agusan del Norte |  |  |  |  |  |  |  | *reference* |
| Agusan del Sur |  |  |  |  | 0.306 | 0.480 | **0.009**** | **0.23 (0.08-0.70)** |
| Surigao del Norte |  |  |  |  | -1.439 | 0.554 | 0.154 | 0.37 (0.10-1.44) |
| Surigao del Sur |  |  |  |  | -0.206 | 0.4­50 | 0.267 | 0.46 (0.12-1.80) |
| **Interaction~ 4Ps*Province** |  |  |  |  |  |  |  |  |
| **4Ps: Agusan del Norte** |  |  |  |  |  |  |  | *reference* |
| 4Ps: Agusan del Sur |  |  |  |  | -0.653 | 0.347 | 0.060 | 0.520 (0.26-1.03) |
| 4Ps: Surigao del Norte |  |  |  |  | 0.561 | 0.357 | 0.115 | 1.75 (0.87-3.53) |
| 4Ps: Surigao del Sur |  |  |  |  | -0.245 | 0.306 | 0.423 | 0.78 (0.43-1.42) |
| **Random effects:** | | | | | | | | |
| **Groups**  **..** | | **Var** | **Std. dev** | **N group** | **Var** | **Std. dev** | **N group** | ***p^a^* Model 2** |
| **Year measured (intercept)**  (Intercept) | | 0.25 | 0.504 | 6 | 0.26 | 0.505 | 6 |  |
| **School (intercept)**  (Intercept) | | 1.05 | 1.024 | 10 | 0.92 | 1.001 | 10 |  |
| **AIC** | | 4616 |  |  | 4609 |  |  | **0.003**** |

*p=* p-value*, ^a^* ANOVA, Est= estimate, SE= standard error, OR= odds ratio, CI= confidence interval, Var= Variance, Std. dev= standard deviation

| **Table S4.** Mixed effects logistic for DBM (concurrent stunting and wasting/thinness or overweight/obesity) in children and adolescents | | | | | | | | |
| --- | --- | --- | --- | --- | --- | --- | --- | --- |
|  | **Model 1_D_** | | | | **Model 2_D_** | | | |
| **Parameter** | **Est.** | **SE** | ***p*** | **OR (CI)** | **Est.** | **SE** | ***p*** | **AOR (CI)** |
| **Fixed effects:** | | | | | | | | |
| Intercept | **-6.077** | **0.585** | **<0.001*** |  | -5.911 | 0.501 | **<0.001*** |  |
| **Inclusion to CCT** |  |  |  |  |  |  |  |  |
| Non-4Ps |  |  |  | *reference* |  |  |  | *reference* |
| 4Ps | **0.058** | **0.197** | 0.770 | 0.94 (0.64, 1.39) | -0.055 | 0.195 | 0.777 | 1.05 (0.72,1.55) |
| **Sex (Female)** | **-0.128** | **0.175** | 0.466 | 0.88 (0.62, 1.24) | -0.132 | 0.176 | 0.451 | 0.87 (0.62,1.24) |
| **Age** | **0.255** | **0.040** | **<0.001*** |  | 0.251 | 0.038 | **<0.001*** |  |
| **Ethnic group** |  |  |  |  |  |  |  |  |
| Major tribes |  |  |  |  | 0.382 | 0.327 | 0.243 | 1.46 (0.77, 2.78) |
| Minor tribes |  |  |  |  | 0.473 | 0.255 | 0.064 | 1.60 (0.97, 2.64) |
| Non-indigenous |  |  |  |  |  |  |  | *reference* |
| **Province** |  |  |  |  |  |  |  |  |
| Agusan del Norte |  |  |  |  |  |  |  | *reference* |
| Agusan del Sur |  |  |  |  | -0.646 | 0.369 | 0.080 | 0.52 (0.25,1.08) |
| Surigao del Norte |  |  |  |  | 1.117 | 0.259 | **<0.001*** | 3.05 (1.84, 5.08) |
| Surigao del Sur |  |  |  |  | -0.990 | 0.398 | **0.013*** | 0.37(0.17, 0.81) |
| **Random effects:** | | | | | | | | |
| **Groups** | | **Var** | **Std. dev** | **N group** | **Var** | **Std. dev** | **N group** | ***p^a^* Model 2** |
| **School (intercept)** | | 0.619 | 0.787 | 10 | 0 | 0 | 10 |  |
| **AIC** | | 1183 |  |  | 1172 |  |  | **<0.001*** |

*DBM= Double Burden of Malnutrition, p=* p-value*, ^a^* ANOVA, Est= estimate, SE= standard error, OR= odds ratio, CI= confidence interval, Var= Variance, Std. dev= standard deviation
