# Supplementary material for: Mixed-method study on the association between inclusion to conditional cash transfer program and the multiple faces of malnutrition in children and adolescents aged 3 to 19 years: a school-based evidence from Caraga Region, the Philippines
Source: BMC Pediatr. 2023 Dec 13;23:630. doi: 10.1186/s12887-023-04438-8 (PMC10717276; doi:10.1186/s12887-023-04438-8)
Supplement: Supplementary file 2 — Additional file 2: Appendix 3. Open codes, focused codes and clusters developed based on the data from the focused group discussions of parents, teachers, 4Ps coordinators and school nurses. Table S5. Sample FGD obtained from teacher participants using open codes, focused codes, and clusters. Table S6. FGD-sample obtained from teacher participants. Table S7. FGD-sample obtained from parent participants under 4Ps. [file 12887_2023_4438_MOESM2_ESM.docx]

**Appendix 3. Open codes, focused codes and clusters developed based on the data from the focused group discussions of parents, teachers, 4Ps coordinators and school nurses.**

| **Table S5.** Sample FGD obtained from teacher participants using open codes, focused codes, and clusters. | | |  |
| --- | --- | --- | --- |
| **Programs and services** | **Why?** | **Nutrition** | |
| **Access to Health Programs and Services to Child’s Nutrition** | | | |
| ***Feeding program** | newly implemented | Fairly adequate | |
|  | dependent on budget allocated |  |  |
|  | perfect attendance | Highly adequate | |
|  | 4Ps & malnourished |  |  |
|  | DepEd-led |  |  |
|  | daily for 120 days |  |  |
|  | 50% more 4Ps |  |  |
|  | most pupils are 4Ps |  |  |
|  | increase height /weight |  |  |
|  | planned menu |  |  |
|  | innovative ways |  |  |
|  | inclusion based on report |  |  |
|  | shy students | Poorly adequate | |
|  | leftovers for non-participants |  |  |
|  | most non-4Ps joined |  |  |
|  | belief of feeding for malnourished only |  |  |
| ***Health center provision** | free/ tastes no good | Poor vitamin in-take | |
|  | cash grant/free |  |  |
| ***School provision & others** | Healthy foods inside school | Highly adequate | |
|  |  |  |  |
|  | With cash grant but ask free medicine from neighbor | Fairly adequate | |
|  | With cash grant but ask free medicine from teacher/school |  |  |

| **Table S6.** FGD-sample obtained from teacher participants | |  |
| --- | --- | --- |
| **Sources** | **Perceptions/Experiences** | **Nutrition** |
| **Food provision and preparation** | | |
| * Parent’s use of cash card | Use as collateral for loan | Poorly adequate |
|  | Use as source for gambling |  |
|  | Inappropriate use |  |
|  | Not used for child's food /nutrition |  |
|  | lack of financial management |  |
|  | schooling for cash only |  |
| * Parent’s use of cash grant with other income (farming, labor, carpentry, vending) | buy vitamins for some beneficiaries | highly adequate |
|  | not enough to meet needs | poorly adequate |
|  | malnourished due to gambling |  |
|  | no food at home |  |
|  | misconception of vitamins as luxury |  |
|  | misconceptions of cash grant as luxury not food for kids |  |
|  | sole source of family subsistence |  |
|  | other purpose not food |  |
| * Parent’s management of cash grant | excluded in feeding program | highly adequate |
|  | small family size |  |
|  | big family with good management |  |
|  | good cash management |  |
|  | use in education |  |
|  | have money to buy foods |  |
|  | small family size under 4Ps |  |
|  | big family | Poorly adequate |
|  | small family not able to manage |  |
|  | going to school without food |  |
|  | going to school without food |  |
|  | not able to sustain small family business |  |
|  | buying other than foods |  |
|  | delay release so not able to buy food |  |
|  | seasonal /no jobs to buy foods for family |  |
| * School policy | Child brought veggies /food from home since: | Healthy food in-take |
|  | no food for sale in school |  |
|  | not allowed to go out in school |  |
|  | Inside school offers healthy food: |  |
|  | fruits sold |  |
|  | better taste |  |
|  | veggies |  |
| * Community food culture (depending on the location) | Producing root crops | Healthy food in-take |
|  | Selling fruits & veggies |  |
|  | Raising livestock & swine for meat |  |

**Table S7.** FGD-sample obtained from parent participants under 4Ps

| **Programs** | **Perceptions/Experiences** | **Nutrition** |  |
| --- | --- | --- | --- |
| **4Ps-Child's Attitude Towards Health Services & Programs** | | |  |
| *Cash grant release | not nutritious but child likes it | Poor food in-take |  |
|  | child's choice |  |  |
|  | child's demands knowing cash is released |  |  |
|  | Eating at home | Good food in-take |  |
|  |  |  |  |
| **Cash Grant /Conditionalities Compliance** | | |  |
| *Health status monitoring & weighing in health center & school | weighing | Highly adequate |  |
|  | weighing every month |  |  |
|  | regular for age 10 |  |  |
|  | compliant to health and educ conditionalities |  |  |
|  | deworming and 80% attendance |  |  |
|  | deworming |  |  |
| *4Ps implementation of Family developmental sessions (FDS) | taught about child's health | Highly adequate |  |
|  | what to do for child's nutrition |  |  |
| **Access to health programs and services** | | | |
| Services & Programs | weighing/giving vitamins | Highly adequate | |
| *Health status monitoring & weighing | house visitation every 6 months |  |  |
|  | house visitation every month |  |  |
|  | easy to approach |  |  |
|  | House visitation by BHW |  |  |
|  | strict health provider |  |  |
|  | highly accessible public hospital |  |  |
|  | afraid to approach provider | Poorly adequate | |
|  | strict health provider |  |  |
|  | strict health provider |  |  |
|  | far public hospital (7km) |  |  |
|  | no access |  |  |
| *Giving of medicine, vitamins, and deworming pills | free | Highly adequate | |
|  | opt for medicine when child is sick |  |  |
| **Food Provision and Preparation** | | | |
| * Food from home | rare meat /salty | Poor food in-take | |
|  | salty food |  |  |
|  | fast & easy to prepare |  |  |
|  | with protein /meat | Good food in-take | |
|  | belief that nutrition is important |  |  |
|  | veggies, fresh fruits, fish, milk, medicine |  |  |
|  | Healthy foods |  |  |
| * Budget allocation (from income) | budget-based purchases | Highly adequate | |
|  | prioritize food for tight budget |  |  |
|  | always wanting / in shortage | poorly adequate | |
|  | fitting the meager budget |  |  |
|  | cheaper foods due to tight budget | Poor food in-take | |
|  | not nutritious but child likes it |  | |
|  | fast/easy to prepare that child likes |  | |
| * Management of cash grant | allocate for vitamins | highly adequate | |
|  | special food like roasted chicken |  |  |
|  | special food like roasted chicken & soda |  |  |
|  | allocate for savings |  |  |
|  | budget for recreation |  |  |
|  | too much if asking for complementary |  |  |
|  | (Happy with this additional cash grant) |  |  |
|  | hoping to be given complementary subsidy |  |  |
|  | budget for vitamins /healthy foods |  |  |
|  | helps a lot |  |  |
|  | prioritize rice for tight budget | poorly adequate | |
|  | budgets lend to non-4Ps parents |  |  |
|  | budget promised to be lent once released |  |  |
|  | budget lent not paid |  |  |
| * Rice subsidy | too much if asking for complementary | highly adequate | |
|  | (Happy with this additional cash grant) |  |  |
|  | hoping to be given complementary subsidy |  |  |
